# Molecular typing reveals the co-existence of two transmission cycles of American cutaneous leishmaniasis in the Andean Region of Venezuela with *Lutzomyia migonei* as the vector

**DOI:** 10.1590/0074-02760180323

**Published:** 2018-12-06

**Authors:** Annhymariet Torrellas, Elizabeth Ferrer, Israel Cruz, Héctor de Lima, Olinda Delgado, José Carrero Rangel, José Arturo Bravo, Carmen Chicharro, Ivonne Pamela Llanes-Acevedo, Michael A Miles, María Dora Feliciangeli

**Affiliations:** 1 Universidad de Carabobo, Facultad de Ciencias de la Salud, Centro Nacional de Referencia de Flebotomos y otros Vectores, Instituto de Investigaciones Biomédicas Dr Francisco J Triana-Alonso, Maracay, Venezuela; 2 Universidad de Carabobo, Facultad de Ciencias de la Salud, Instituto de Investigaciones Biomédicas Dr Francisco J Triana-Alonso, Maracay, Venezuela; 3 WHO Collaborating Centre for Leishmaniasis, National Center for Microbiology, Instituto de Salud Carlos III, Majadahonda, Madrid, Spain; 4 Ministerio del Poder Popular para la Salud, Servicio Autónomo, Instituto de Biomedicina, Caracas, Venezuela; 5 Universidad Central de Venezuela, Instituto de Medicina Tropical, Caracas, Venezuela; 6 Servicio de Dermatologia, Municipio Tovar, Merida, Venezuela; 7 Faculty of Infectious and Tropical Diseases, London School of Hygiene and Tropical Medicine, Department of Pathogen Molecular Biology, London, United Kingdom

**Keywords:** Leishmania, epidemiology, diagnosis, PCR-RFLP

## Abstract

BACKGROUND The transmission routes for American cutaneous leishmaniasis (ACL) are in flux, so studies examining its transmission in humans, mammalian hosts, and sand fly vectors are urgently needed. OBJECTIVES The aim of this work was understand the epidemiological cycles of *Leishmania* spp., which causes ACL in the Andean Region of Venezuela, by identifying the *Leishmania* and the sand fly species involved in human and dog infections. METHODS Thirty-one biopsies from patients in Mérida and Táchira states with suspected ACL were studied by both parasitological tests (cultures and hamster inoculation) and a molecular test [Internal transcribed spacer 1 (ITS1) nested polymerase chain reaction-restriction fragment length polymorphism (PCR-RFLP)]. We also conducted a survey to detect *Leishmania* infection in dogs (Immunifluorescence antibody test and ITS1 nested PCR-RFLP) and sand flies (ITS1 nested PCR-RFLP) from El Carrizal, a highly endemic focus of ACL in Venezuela. FINDINGS Three different *Leishmania* species were identified in the clinical samples from humans (*Leishmania braziliensis*, *L. guyanensis*, and *L. mexicana*) and dogs (*L. guyanensis* and *L. mexicana*). The predominant sand fly species found were those from the Verrucarum group (infected with *L. mexicana*) and *Lutzomyia migonei* (infected with *L. guyanensis* and *L. mexicana*). MAIN CONCLUSIONS We show that *Lu. migonei* may be the putative vector in two ACL epidemiological cycles, involving *L. guyanensis* and *L. mexicana*. We also report for the first time the presence of *L. guyanensis* in domestic animals.

Molecular epidemiology can unravel the complexities of transmission cycles, thereby providing guidance for the control strategies used to manage vector-borne diseases.^1^ The polymerase chain reaction-restriction fragment length polymorphism assay (PCR-RFLP), based on an analysis of the ribosomal DNA internal transcribed spacer 1 (ITS1) sequence, has increasingly been used to identify *Leishmania* spp., because almost all the medically relevant *Leishmania* parasites from different endemic regions can be identified by this technique.^2^ PCR-RFLP, or variants of it, has been used to detect and identify the different *Leishmania* spp. that cause American cutaneous leishmaniasis (ACL).^3^

Several studies have reported the presence of ACL in dogs.^4^ ^,^ ^5^ ^,^ ^6^ The detection of leishmanial DNA in canine samples has gained attention both in the diagnosis of ACL in dogs and in epidemiological studies.^7^

Molecular tools have also helped detect and identify *Leishmania* spp. in phlebotomine sand flies.^8^ ^,^ ^9^ ^,^ ^10^ However, due to the criteria needed to incriminate a sand fly species as vector, one should be cautious when interpreting Leishmania-positive PCR results in sand flies. Killick-Kendrick^11^ suggested that the following criteria should be fulfilled in order to consider a sand fly species as a vector: (i) evidence for the overlapping geographical distribution of the vector and the human disease, (ii) the vector feeds on humans, and (iii) the vector supports natural gut infections with promastigotes of the same *Leishmania* species that occurs in humans. Based on the above criteria, about 530 sand fly species exist on the American continent,^12^ 56 of them belonging to the genus *Lutzomyia* that are suspected or proven vectors responsible for sustaining one or more ACL epidemiological cycles.^13^

The main criticism used by classical entomologists against the use of molecular tools is that the detection of parasitic nucleic acids does not prove the presence of live, infective organisms (metacyclic forms) in the vectors. However, the ability to readily apply such molecular methods to all components of the transmission cycle (human, animal reservoir, and vector) makes a fundamental contribution to our understanding of the epidemiology of leishmaniases.

In Venezuela, only a few epidemiological studies have been conducted, especially in the Andean Region. Between 2003 and 2007, Mérida state reported an ACL incidence rate of 19.43/100,000 inhabitants.^14^ The Dermatology Service in the municipality of Tovar (184 km^2^; 35,000 inhabitants) recorded 93 cases (11.85% of the total), with the majority of them (24.73%) arising from the village of El Carrizal. Based on this background and logistical facilities, during 2008 - 2009 we carried out the detection, isolation, and identification of *Leishmania* parasites from patients attending the Municipal Dermatology Service with dermal lesions compatible with ACL, from dogs, and from sand flies in the village of El Carrizal.

## MATERIALS AND METHODS

**Fig. 1:**
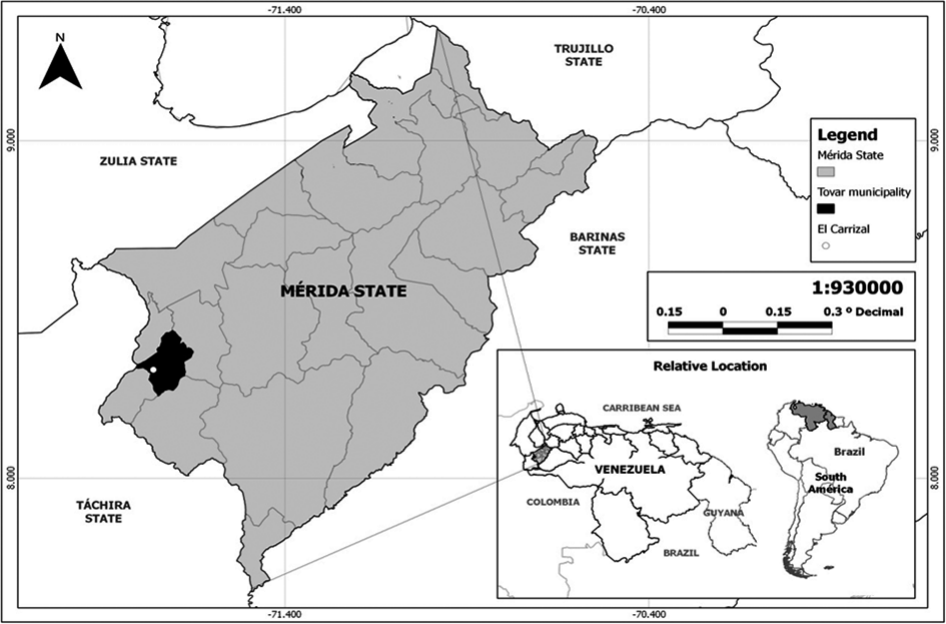
map of the Venezuelan Andean Region highlighting Mérida state and the municipality studied.

*Ecological framework* - The landscape of the Venezuelan Andean Region, which mainly includes the states of Trujillo, Merida, and Táchira, as well as the highlands of Barinas and Apure states, encompasses a cloudy high mountain forest, moorland, and a cloudy lowland tropical rainforest. The climate is bimodal, with a rainy season and a dry season; the main rains occurring between the months of April and May, and during the months of September to November, both with average monthly rainfall exceeding 120 mm, whereas in other months, rain is scarce. The average annual precipitation, temperature (T), and relative humidity (RH) are, 1891 mm, 19.5ºC ,and 78%, respectively (data registered by the Venezuelan Air Force, VAF). The studied village was El Carrizal which is located at 08º17´63”N, 71º45’75”W (Fig. 1).

*Human samples* - From January to December 2008, 61 patients attended the Dermatology Service with lesions compatible with ACL; 31 patients consented to sampling from the lesion. Twenty-five of the patients came from Merida state, and six from the bordering Andean Táchira state. A member of the study team (JCR), a medical doctor and head of the Dermatology Service, performed the skin biopsies. Samples were placed into polypropylene cryotubes with 10% dimethyl sulphoxide (DMSO), and labelled to allow tracking of the patient, location, and date. The tubes were stored at -196ºC in aluminium canes submerged in liquid nitrogen in a cryological tank^15^ to be transported to the BIOMED laboratory (Universidad de Carabobo, Maracay) for parasitological and molecular tests.

*Canine survey* - In 2008, El Carrizal had a population of 624 inhabitants, living in 152 dwellings. Their main occupation was agriculture (cereals and legumes, roots and tubers, vegetables, bananas and coffee). Tourism and commerce were other important activities.

Sixty-eight out of the 152 families (44.73%), almost proportionally distributed across four sectors of the village, were visited and informed about cutaneous leishmaniasis, the risk of having infected dogs in the house, and the objectives of our research. Informed consent to take blood samples from the animals was obtained from 43 houses, each with up to five dogs; the other 25 dwellings having no dogs. A total of 69 dogs were registered (without any sign of lesions, or scars compatible with ACL), but only 28 of the dogs (40.58%) were sampled, due to limiting factors, such as absence of the dogs from the house during site visits, aggressive dogs that were difficult to handle, or roaming dogs that could not be located. Blood samples were transported to the laboratory in polystyrene containers with freezing packs to allow for the molecular detection and identification of parasites.

*Sand fly survey* - For the purpose of this epidemiological study, monthly sampling collections were carried out from January 2008 to January 2009, we selected those specimens that were collected during January-March 2008 and November 2008-January 2009, as these months correspond to the peaks of the sand fly population, and which would include nulliparous and parous sand flies. Trapping was conducted in five houses spaced across the village whose owners agreed to collaborate. Three of these houses reported 8, 2, and 11 cases of ACL. Three CDC light traps (John W. Hock Company, Gainesville, Florida, USA) were placed overnight (from 18:00-19:00 to 6:00-7:00) in each house for three-four consecutive days per month.

One trap was located indoors in the main sleeping room or the room adjacent to it. A second trap was placed outdoors (0-20 metres from the house) and close to the resting places of the domestic animals, predominantly dogs or chickens. The third trap was located in the woodland, approximately 100 metres from the houses. Additionally, when the weather was favourable, a Shannon trap was also placed in the woodland, between 19:00 to 22:00, further from the CDC trap (10 metres). All the sand flies collected were stored in vials containing absolute ethanol, and labelled to allow tracking of collection date, trap number, house, and habitat.

Identification of males and females sand flies was carried out in the laboratory based on morphological characters described in the guide to the identification of *Lutzomyia sandflies* of Young and Duncan.^16^ A quick and reliable method for the large-scale identification of females and *Leishmania* spp. was used to separate females into pools by species, date, trap, house and habitat.^17^ Briefly, each pool was washed three times with distilled water, before placing each specimen in a small drop of diluted phenol (40%) on a microscope slide (usually ten sand flies per slide) to allow for rapid clarification. The sand flies were identified under 250 X and 400 X magnifications based on morphological characters (genitalia, genital pump, genital filaments and the aedeagus in males, and pharynx, cibarium, horizontal and vertical teeth, spermathecae, and spermathecal ducts in females). Each female was then washed again four times in distilled water, and then pooled according to the trap, house of origin, and species, with a maximum of 20 sand flies per vial in Eppendorf tubes containing lysis buffer (0.02 M NaCl, 0.5 M EDTA pH 8.0, 1M Tris-HCl, pH 7.4) and stored at 4ºC. Blood-fed females were kept apart for a separate study on the identification of blood-meal sources (in preparation).

*Parasitological tests* - With the aim of isolating the parasites, a portion of each of the biopsies from patients with suspected ACL was cultured in liver infusion tryptose (LIT) culture medium supplemented with 20% inactivated foetal bovine serum, and in Novy-McNeal-Nicolle medium. Cultures were replicated every 8-10 days, and observed for parasite growth, and they were discarded if they remained negative after four replicate cultures have been performed. In addition, another portion of the biopsy was macerated in sterile PBS and inoculated into the footpads of hamsters (*Cricetus auratus*), which were checked weekly for two months to detect infection, with the aim of isolating the parasites.

*Immunofluorescence antibody test* - We used an immunofluorescence antibody test (IFAT) to detect the presence of antibodies against *Leishmania* spp. in the sera from dogs. The antigen was prepared according to the method described by Pappas et al.,^18^ using whole *Leishmania* promastigotes grown *in vitro* from isolates obtained from cutaneous lesions of patients with ACL, who were diagnosed and treated at the “Laboratorio de Inmunodiagnóstico of the Instituto de Medicina Tropical, Universidad Central de Venezuela”. The reaction was considered positive when more than 50% of the parasites showed complete peripheral fluorescence (in titres > 1:16).

*Molecular tests* - For the detection and identification of *Leishmania* spp., all the samples from patients, dogs, and sand flies, either fed or unfed, were analysed by the PCR-RFLP method using the ribosomal DNA internal transcribed spacer 1 (ITS1) as the target sequence.

DNA was extracted from skin biopsy samples obtained from human patients, as well as blood samples from dogs and sand fly pools. The sand fly pools used for PCR-RFLP were selected randomly, taking into account the different species, areas, and types of capture represented, and a Proteinase K- phenol-chloroform extraction was performed as previously described.^19^ DNA pellets were dried and then re-dissolved in 50 µL of sterile distilled water. The samples were kept at 4ºC until analysis. DNA was also extracted from cultures of the following *Leishmania* reference strains: *L.* (*V*) *braziliensis* MHOM/BR/1975/M2903, *L.* (*V*) *guyanensis* MHOM/BR/1975/ M4147, and *L.* (*L*) *mexicana* MHOM/BZ/1982/BEL21.

The molecular test for ITS-1 nested PCR - *Hae*III RFLP was performed according to protocols described by Schönian et al.^2^ and Cruz et al.,^20^ with primers LITSR (5’CTGGATCATTTTCCGATG3’) and L5.8S (5’TGATACCACTTATCGCACTT3’) for the first amplification, and SAC (5’CATTTTCCGATGATTACACC3’) and VAN2 (5’GCGACACGTTATGTGAGCCG3’) for the second amplification. The PCR products were digested with the enzyme *Hae*III according to the manufacturer’s protocol*.* Restriction fragments were subjected to electrophoresis in 2% agarose at 100V in 0.5-TBE (0.045 M Tris-borate, 1 mM EDTA) buffer and visualised under ultraviolet light after staining for 15 min with ethidium bromide (0.5 µg/mL). Negative controls were the reaction mixture and water; positive controls were reference strains of the most common species of *Leishmania* spp. circulating in the area.

**TABLE I:** Efficiency of *Leishmania* detection tests and species identification on skin biopsies from patients with suspected American cutaneous leishmaniasis (ACL), Mérida and Táchira states

Technique	(Nº)	(%)	*L. braziliensis*	*L. guyanensis*	*L. mexicana*
Cult/Hams/PCR-RFLP	8	25.81	5	3	0
Culture/PCR-RFLP	7	22.58	4	3	0
Hamster/PCR-RFLP	2	6.45	1	0	1
PCR-RFLP	8	25.81	1	3	4
Negative	6	19.35	-	-	-
Total	31	100.00	11	9	5

Cult: positive by parasite culture; Hams: positive by hamster inoculation; PCR-RFLP: positive by polymerase chain reaction-restriction fragment length polymorphism (PCR-RFLP); N°: the number of samples positive to the parasite according to the detection test; % represent the efficiency of the method to detect the parasites.

*Statistical analysis* - Data for the study were recorded using Microsoft Excel 2010. The frequency distributions and percentages were calculated for all collected variables. Tests for homogeneity (by Chi square, *X* ^*2*^ ) were calculated to compare the proportions of infection by culture, hamster inoculation (xenodiagnoses), IFAT, and PCR-RFLP, and also to compare the distribution of *Leishmania* species. The significance level was 0.05 with confidence limits (CL) of 95%.

*Ethical considerations* - The project was approved by the Committee of Bioethics of the Institute of Biomedical Research of the University of Carabobo (BIOMED-UC), following the guidelines for the care of humans and animals issued by the Commission of Bioethics of the Ministry of Science and Technology and the Operational Guidelines for Ethics Committees that Review Biomedical Research (TDR/PRD/ETHICS/2000.1). Field work was carried out in close collaboration with the Service of Dermatology of the Tovar Municipality, and the samples from patients were taken and provided according to the protocols of the Control Program of Leishmaniasis. Informed consent was requested and signed by the owners of dogs from which blood samples were taken, according to the applicable ethical regulations.

**Fig. 2:**
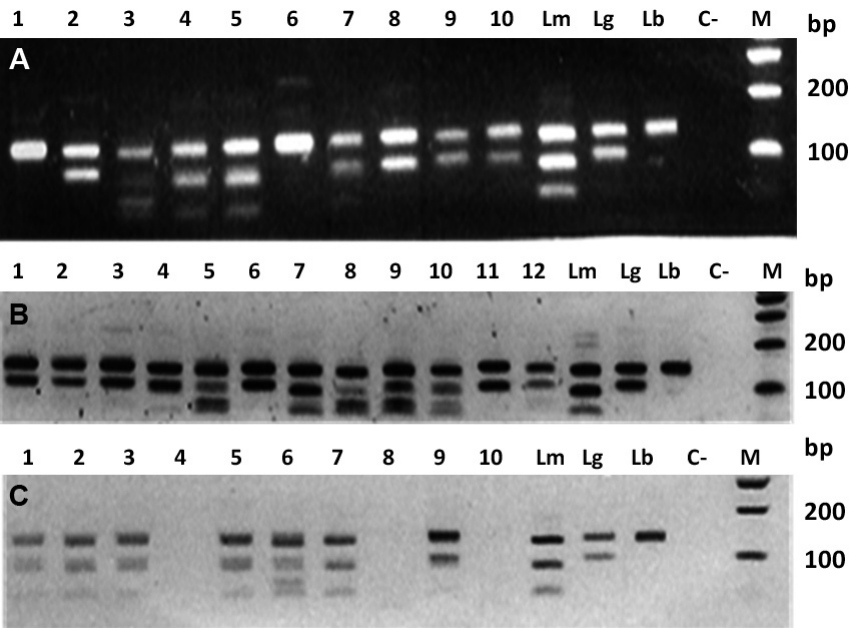
molecular typing of *Leishmania* isolates from human, dogs and sand flies. The *Hae*III digested polymerase chain reaction (PCR) products were analysed by electrophoresis and ethidium bromide staining in 2% agarose gel. Lanes Lm: *L. mexicana* (MHOM/BZ/1982/BEL21); Lg: *L. guyanensis* (MHOM/BR/1975/M4147); and Lb: *L. braziliensis* (MHOM/BR/1975/M2903). Lane C-: negative control; Lane M: molecular weight marker (100 bp ladder, Promega). (A) Lanes 1-10: positive human clinical samples; (B) Lanes 1-12: positive canine samples; (C) Lanes 1-3, 5-7 and nine positive *Lutzomyia migonei* pools.

## RESULTS

*Human samples* - Table I shows the results of the parasitological and molecular tests from 31 biopsies of patients with suspected ACL. As a result, 8/31 (25.81%) were positive by all three techniques (culture, hamster inoculation, and PCR-RFLP), 7/31 (22.58%) were positive by culture and PCR-RFLP, 2/31 were positive by hamster inoculation and PCR-RFLP (6.45%), 8/31 (25.81%) were positive only by PCR-RFLP, and 6/31 (19.35) were negative by all techniques. No statistical difference (χ^2^ = 1.68; df = 1; p = 0.195) was observed between the proportion of positive parasite cultures (48.4%), and positive xenodiagnoses (32.2%) (χ^2^ = 1.68, df = 1; p = 0.195), but PCR-RFLP positivity (80.6%) was significantly higher than a positive xenodiagnosis (χ^2^ = 14.76, df = 1; p < 0.001), as well as a positive culture (χ^2^ = 7.05, df =1; p = 0.007).

An analysis of the PCR-*Hae*III*-*RFLP of the 25 PCR positive samples showed that three species of *Leishmania* were responsible for causing ACL in these patients: *L. braziliensis* in 11 cases, *L. guyanensis* in nine cases, and *L. mexicana* in five cases (Table I); Fig. 2A shows the PCR-RFLP results. A chi squared homogeneity test showed that there was no statistical difference in the distribution of *L. braziliensis*, *L. guyanensis*, and *L. mexicana* in the screened samples (p = 0.2634).

**TABLE II:** Results of immunofluorescence antibody test (IFAT), polymerase chain reaction (PCR) and *Leishmania* identification by PCR-restriction fragment length polymorphism (PCR-RFLP) on dogs blood samples from the American cutaneous leishmaniasis (ACL) focus El Carrizal, Mérida state, in the Andean Region of Venezuela

IFAT	PCR	N	IFAT + %	PCR + %	*L. guyanensis*	*L. mexicana*
IFAT + (1/16)	PCR +	5	17.86	17.86	3	2
IFAT + (1/32)	PCR +	5	17.86	17.86	5	0
IFAT + (1/32)	PCR -	2	7.14	-	-	-
IFAT + (1/64)	PCR +	6	21.43	21.43	3	3
IFAT -	PCR +	9	-	32.14	6	3
IFAT -	PCR -	1	-	-	-	-
Total (n) %		(28) 100	(18) 64.29	(25) 89.29	(17) 68	(8) 32

Numbers in parentheses: titers of antibodies. Cut off point, titers ≥ 1/16.

*Dog samples* - Eighteen of the 28 dogs evaluated were positive by IFAT (64.29%), and 25 were positive by PCR (89.29%) (Table II), with PCR positivity being significantly higher than IFAT positivity (χ^2^ = 4.91, df =1; p = 0.026). Seventeen (68%) of the PCR-positive dogs were infected with *L. guyanensis*, and eight (32%) with *L. mexicana* (Table II, Fig. 2B).

*Sand flies* - A total of 4,786 female and 2,514 male sand flies were collected in El Carrizal (Table III). The predominant species found were those from the Verrucarum group, possibly *Lutzomyia youngi* and *Lu. spinicrassa*, as revealed by the males found, since the females are indistinguishable by morphological characters. *Lu. migonei* was also quite frequent (377 females, 980 males), whereas other species were seldom trapped.

*Leishmania spp. infection in sand flies -* A random sample of females was selected for the preparation of pools for *Leishmania* spp. detection and typing, according for differences in species, capture area, and capture method. Pools included 2,636 females from the Verrucarum group, 217 *Lu. migonei*, 21 *Lu. lichyi*, and 21 *Lu. nuneztovari* females. Results of the *Leishmania* infection in these species, as detected by PCR-RFLP per pools, and per the habitat of collection, are shown in Table IV (Fig. 2C). Females of both the Verrucarum group and *Lu. migonei* were caught and found infected in three habitats: indoors, outdoors, and in the woodlands. No positive PCR was obtained from the pools of *Lu. lichyi* and *Lu. nuneztovari*.

*Minimum natural infection rate (MNIR)* - Considering that at least one specimen was infected in each of the PCR-positive pool, the minimum natural infection rate in the Verrucarum group would be 0.34% [nine pools identified as *L. mexicana* *(100/total females tested in the positive and negative pools) (9*(100/2,636)]. For *Lu. migonei* the MNIR for *L. mexicana* would be 4.15% [9*(100/217)], and 0.46% for *L. guyanensis* [1*(100/217)].

## DISCUSSION

The aim of this work was to contribute to the knowledge of the epidemiological cycles of *Leishmania* spp., which is a cause of ACL in the Andean Region of Venezuela. The methods commonly used to diagnose ACL according to the guidelines of the Ministry of Health, are the leishmanin skin test (LST), as a triage method, that is used in all the Regional Dermatology Services.^14^ and whenever possible, the isolation of parasite using LIT-NNN culture and microscopy for confirmation. However, molecular tools have the additional advantage of increased sensitivity, and allow for definitive species identification. In previous studies in Mérida and Táchira states, both *L. braziliensis* and *L. mexicana* have been confirmed as being causative agents for ACL.^21^ In this study, we confirmed those findings for *L. brazilienis* and *L. mexicana*, and further report the involvement of *L. guyanensis* in ACL in this region for the first time. In Andean countries, *L. peruviana* is the principal species that causes ACL in Peru, whereas in Ecuador the principal causative species is *L. mexicana*.^6^

We also confirmed *Leishmania* infection and exposure in dogs from El Carrizal, Mérida state by PCR (89.3% positive) and IFAT (64.3%) respectively, pointing to the role of the dog as reservoir of ACL etiological agents.

Similar findings have been reported in other ACL regions. In Brazil, studies by Falqueto et al.^22^ and Madeira et al.^23^ ^,^ ^24^ demonstrated the presence of *L. braziliensis* in cutaneous lesions, and *L .chagasi*, isolated from different sites, in the same animal. However, some studies indicate that humans, and not dogs, are probably the most important domestic reservoirs of *L. braziliensis*.^25^ In a study conducted in Peru,^26^ found that 81% and 31% of the dogs were positive by ELISA and PCR respectively, which suggests the potential role of dogs as reservoirs hosts. Calzada et al.^27^ also observed a high positive rate by ELISA (47%) in dogs in Trinidad de las Minas, Panamá, whilst they were not able to detect any positive dogs by PCR. Nevertheless, the infection of asymptomatic dogs with sand flies in ACL endemic regions has been demonstrated by Rojas and Scorza,^28^ who confirmed the transmission of *Leishmania* spp. to reared *Lu. youngi* sand flies (0.88% infection rate) by xenodiagnosis using dogs from an endemic area in Trujillo, Venezuela.

Here, we have demonstrated the value of the PCR to reveal cryptic infections by *L. guyanensis* and *L. mexicana* in dogs from an ACL endemic area, revealing interesting changes in the classical *L. guyanensis* epidemiological cycle normally associated with the Amazon rain forest and or sylvatic transmission, instead of domestic animals hosts. The absence of *L. braziliensis* in the dogs that we sampled suggests that, although dogs may still be a reservoir, infections might be acquired from a sylvatic source, since all the New World cutaneous leishmaniasis cycles are predominantly zoonotic.^13^ Further studies of the DNA sequences of the *Leishmania* parasites present in the study samples are necessary to gain a better understanding of these findings.

Regarding the ACL vectors in the Andean Region of Venezuela, we found natural infections by *L. mexicana* and *L. guyanensis* in *Lu. migonei*, and by *L. mexicana* in females in the Verrucarum group*.* Naturally infected sand flies were caught in all the habitats studied (indoors, outdoors, and woodland), *Lu. migonei* being more associated with the peri-domestic environment, as has also been found in other countries such as Brazil,^29^ and Argentina.^30^ As shown in a previous study, females in the Verrucarum group were collected in larger numbers indoors compared to outdoors,^31^ which suggests that the transmission of *L. mexicana* occurs frequently in the domestic environment.

**TABLE III:** Species composition and abundance of phlebotomine sand flies collected at El Carrizal, Mérida state, Venezuela

	♀	(%)	♂	(%)
Verrucarum group	4353^*^	90.95		
*Lutzomyia youngi* ^**^		-	1373	54.61
*Lu. spinicrassa* ^**^		-	139	5.53
*Lu. sauroida* ^**^		-	2	0.08
*Lu. migonei*	377	7.88	980	38.98
*Lu. lichyi*	34	0.71	4	0.16
*Lu. nuneztovari*	21	0.44	14	0.56
*Lu. venezuelensis*	1	0.02	0	0
*Brumptomyia beaupertuyi*	0	0.00	2	0.08
Total	4786	100,00	2514	100,00

*: females of the Verrucarum group morphologically indistinguishable; **: males of the Verrucarum group present in the area.

**TABLE IV:** *Leishmania* identification by polymerase chain reaction-restriction fragment length polymorphism (PCR-RFLP) in phlebotomine sand flies collected at El Carrizal, Mérida state

	Habitat (trap)	Neg pools	N° ♀	PCR + pools	N° ♀	Pools RFLP + (N° ♀)	*Leishmania* spp*.*
Verrucarum	Indoors (CDC)	23	429	2	40	2 (40)	*L. mexicana*
Group	Outdoors (CDC)	28	501	3	60	1 (20)	*L. mexicana*
						2 (40)	No identified
	Woodland (CDC)	8	137	0	0	0	
	“ “ (Shannon)	68	1350	7	110	6 (110)	*L. mexicana*
					9	1 (9)	No identified
Total ♀ = 2417 + 219 = 2636		127	2417	12	219	9 (170)	*L. mexicana*
*Lutzomyia migonei*	Indoors (CDC)	0	0	3	60	3 (60)	*L. mexicana*
	Outdoors (CDC)	0	0	6	84	4 (56)	*L. mexicana*
	“ “ “	0	0			1 (8)	*L. guyanensis*
	“ “ “	0	0			1 (20)	No identified
	Woodland (CDC)	2	29	1	20	1 (20)	*L. mexicana*
	“ “ (Shannon)	1	20	2	4	1(4)	*L. mexicana*
		3	49	12	168	9 (140)	*L. mexicana*
Total ♀ = 49 +168 = 217						1 (8)	*L. guyanensis*

CDC: CDC light traps; neg pools: pools of negative sand flies by PCR; PCR + pools: pools of positive sand flies by PCR; pools RFLP +: pools with *Leishmania* identification by RFLP.

The first record of the natural infection of sand fly species by *Leishmania* spp. promastigotes in Venezuela was made by Pifano and Ortiz (1952),^32^ ^)^ who reported *Leishmania* spp. infection in *Lu. migonei*. To the best of our knowledge, no more data have been published on this subject until our preliminary results on the role of this species in the transmission of ACL were reported at El Carrizal (Feliciangeli et al. 2011).^33^

The presence of *Lu. migonei* has also been recorded in Colombia, Trinidad and Tobago, Argentina, Bolivia, Brazil, Ecuador, Paraguay, and Peru.^34^ However, records of a natural infection by *Leishmania* spp. causing ACL are only available from Brazil, where infection by *L. braziliensis* in *Lu. migonei* was documented in specimens collected in Northeastern Brazil, in the Baturité hills,^35^ and in Jacarepaguá, Rio de Janeiro.^8^ Subsequently, Carvalho et al.^9^ reported similar findings for *Lu. migonei* specimens collected in Praia Vermelha, Ilha Grande, and Rio de Janeiro*.* In Argentina, *Lu. migonei*, is thought to be the vector for ACL, due to the presence of *L. braziliensis* in several foci,^36^ as well as of human and canine visceral leishmaniasis in a rural focus, less than 10 km distant from Puerto Iguazú City, Misiones, where *Lu. longipalpis* is apparently absent.^30^ Moya et al.^10^ also found *L. infantum* infected *Lu. migonei* in a neighbouring region.

With respect to the species in the Verrucarum group, *Lu. youngi* (= *Lu. townsendi*), has been documented in Costa Rica, Venezuela, and Colombia, whereas *Lu. spinicrassa* seems to be restricted to Venezuela and Colombia.^34^ At El Carrizal we identified a natural infection by *L. mexicana* in these isomorphic species. In Venezuela, in the allopatric ACL focus of Las Calderas, Trujillo, peripyloric promastigotes were seen in *Lu. youngi,* and were thought to be *L. braziliensis* based on the criteria of Lainson and Shaw.^37^ Subsequently, in an extensive work carried out in 21 localities of Mérida state at different altitudes, Añez et al.,^38^ found that among 17 parous sand flies that were dissected, there was a natural *Leishmania* spp. infection in 45% of the *Lu. youngi*, the dominant species at high altitudes, in 9% of *Lu. spincrassa*, a species only found at median altitudes, and in 15% of *Lu gomezi*, a species only found at low altitudes. They concluded that, because of its abundance at > 800 metres above sea level and its high degree of endophagy, as reported by Rojas and Scorza,^28^ *Lu. youngi* is a major vector in the Venezuelan Andes. In Colombia, *Lu. spinicrassa* has been proven to be a *L. braziliensis* vector because of the massive infection by promastigotes in the pylorus and midgut in 1 out of 1,679 cryopreserved and dissected females (0.03%) collected in an allopatric population near Arboledas, north of Santander, and close to Cucuta city^15^ at the border of Táchira state in Venezuela. This species was also confirmed to be infected by *L. braziliensis* in specimens captured in the village of Catarnica, an ACL endemic focus in Táchira state.^39^

In our study we found sand flies infected in three different habitats, thus we can conclude that the transmission of ACL may occur in all of them. Moreover, we discovered the co-existence of two transmission cycles of ACL involving *Lu. migonei* as the vector and the dog as a domestic reservoir of both *L. mexicana* and *L. guyanensis*. Concerning the two species in the Verrucarum group, both *Lu. youngi* and *Lu. spinicrassa* might contribute to the transmission of *L. mexicana*. We have not as yet determined if any of these three sand fly species act as local vectors for *L. brazilensis.* Further studies with the approaches that we have employed will clarify the detailed dynamics of *Leishmania* spp. transmission in the ACL endemic foci of the Andean Region of Venezuela, and inform improved strategies for disease control.
